# Covid-19 epidemic in Italy: evolution, projections and impact of government measures

**DOI:** 10.1007/s10654-020-00631-6

**Published:** 2020-04-18

**Authors:** Giovanni Sebastiani, Marco Massa, Elio Riboli

**Affiliations:** 1 Istituto per le Applicazioni del Calcolo “Mauro Picone”, Consiglio Nazionale delle Ricerche, Via dei Taurini 19, 00185 Rome, Italy; 2 Department of Mathematics Guido Castelnuovo, Sapienza University, Piazzale Aldo Moro 5, 00185 Rome, Italy; 3 Department of Mathematics, Imperial College London, 16-18 Princes Gardens, London, SW7 1BA UK; 4 Medical School, Humanitas University, Via Rita Levi-Montalcini, Pieve Emanuale, 20090 Milan, Italy; 5 School of Public Health, Imperial College London, Norfolk Place, London, W2 1PG UK

**Keywords:** COVID-19, Epidemic, Italy, Cumulative incidence, Growth rate, Projections

## Abstract

We report on the Covid-19 epidemic in Italy in relation to the extraordinary measures implemented by the Italian Government between the 24th of February and the 12th of March. We analysed the Covid-19 cumulative incidence (CI) using data from the 1st to the 31st of March. We estimated that in Lombardy, the worst hit region in Italy, the observed Covid-19 CI diverged towards values lower than the ones expected in the absence of government measures approximately 7–10 days after the measures implementation. The Covid-19 CI growth rate peaked in Lombardy the 22nd of March and in other regions between the 24th and the 27th of March. The CI growth rate peaked in 87 out of 107 Italian provinces on average 13.6 days after the measures implementation. We projected that the CI growth rate in Lombardy should substantially slow by mid-May 2020. Other regions should follow a similar pattern. Our projections assume that the government measures will remain in place during this period. The evolution of the epidemic in different Italian regions suggests that the earlier the measures were taken in relation to the stage of the epidemic, the lower the total cumulative incidence achieved during this epidemic wave. Our analyses suggest that the government measures slowed and eventually reduced the Covid-19 CI growth where the epidemic had already reached high levels by mid-March (Lombardy, Emilia-Romagna and Veneto) and prevented the rise of the epidemic in regions of central and southern Italy where the epidemic was at an earlier stage in mid-March to reach the high levels already present in northern regions. As several governments indicate that their aim is to “push down” the epidemic curve, the evolution of the epidemic in Italy supports the WHO recommendation that strict containment measures should be introduced as early as possible in the epidemic curve.

## Introduction

On the 23rd and 24th of February, the Italian National Health Service reported two hot spots of Covid-19 cases in two small geographical areas of northern-Italy located in the Lombardy and Veneto regions. The Italian government promptly reacted by establishing two “red zones” where stringent measures to contain the epidemic were introduced from the 24th of February. These measures included quarantining of the areas, imposing strict restrictions of people movements and the temporary closure of schools, shops and industrial activities. On the 8th of March the government decided to extend these extraordinary measures to all Lombardy and neighbouring provinces, and eventually to the whole country on the 11th of March 2020.  In both instances, the implementation started the day after of the governmental decrees. The Italian Government subsequently took additional measures to further restrict people movement, travel, non-essential industrial activities and social interactions [1].

After continuous growth for nearly 4 weeks, the reported daily number of new Covid-19 cases in Italy stabilized from the 22nd to 31st of March suggesting that the epidemic may have started to slow. However, it is uncertain to what extent governmental measures may have had an impact to takle the spread of the epidemic and what could be the possible time lag between the implementation of these extraordinary measures and the first signs of effectivness.

To address these questions, we analysed the evolution of the Covid-19 cumulative incidence (Covid-19 CI) data and report results on the epidemic curve in relation to the dates of the governmental measures. Moreover, we present a two-month projection of the evolution of the epidemic curve in Lombardy.

## Materials and methods

We analysed the time sequence of the cumulative number of cases for the 107 Italian provinces reported by the Italian Civil Protection using a publicly accessible database [2]. We included in our analyses the data on the number of Covid-19 cases registered from the 1st to the 31st of March 2020, when the Italian Government guidelines indicated that Covid-19 PCR testing should be restricted to symptomatic patients only. Therefore the data used in these analyes include only symptomatic patients who had tested positive at the Covid-19 PCR.

We modelled the curves of the Covid-19 CI for each of the provinces and derived their growth rate to assess whether and when such rate reached a peak. We also computed and analysed the Covid-19 CI in Lombardy, Emilia-Romagna and Veneto, the three regions where the epidemic was initially concentrated and in three regions representative of the epidemic in the centre and south of Italy, namely Tuscany, Campania and Sicily. Firstly, we described the observed cumulative number of cases at both provincial and regional levels using a logistic function model [3].

Subsequently, based on the entire observed sequence of cases we generated the projected evolution of the Covid-19 CI and of the daily rate of new cases up to the 24th of May. The Covid-19 CI was described by a compartment model [3] quantified by a system of ordinary differential equations. This model takes into account the presence of asymptomatic cases. In addition, it incorporates the effect of the governmental interventions by allowing the reproductive number Ro to decrease over time since the start of the government intervention. We also estimated the evolution of the epidemic under the assumption of no government intervention by assuming a constant Ro. In both cases, a Bayesian approach was built, allowing to take into account a priori information about relevant variables, e.g. incubation time.  This also provides both a more robust estimation and confidence intervals. Statistical inference on the unknown model parameters was drawn by means of Markov chain Monte Carlo (MCMC) simulations [4].

## Results

**Fig. 1:**
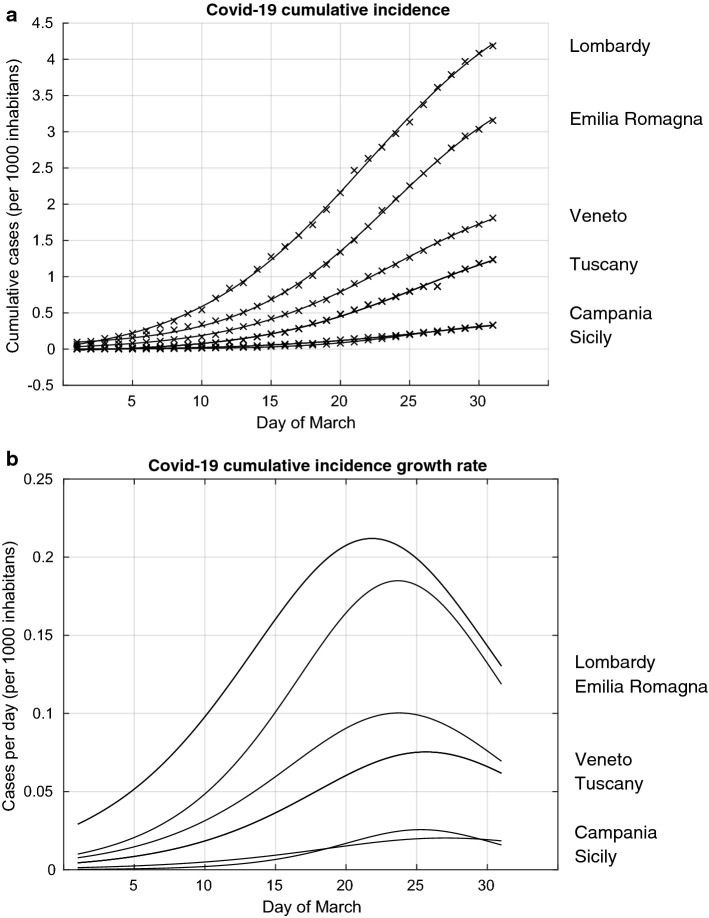
Covid-19 cumulative incidence and cumulative incidence growth rate from the 1st to the 31st of March in six regions of Italy: Lombardy, Emilia-Romagna, Veneto,Tuscany, Campania and Sicily. **a:** Observed cumulative number of cases per 1000 inhabitants (represented by crosses) and the estimated model of the cumulative incidence (continuous line); **b:** estimated cumulative incidence rates per 1000 inhabitants

We present in Fig. 1 the results of the analyses of the observed Covid-19 cumulative incidence (Covid-19 CI) in Lombardy, Emilia-Romagna and Veneto, the three regions where the Covid-19 epidemic initially started and in three regions representative of the centre and the south of Italy, namely Tuscany, Campania and Sicily, where the epidemic started developing later. In Fig. 1a we present both the observed Covid-19 CI and the modelled Covid-19 CI curve using the logistic setting. The highest Covid-19 CI, normalised for 1000 inhabitants, was observed in Lombardy, followed by Emilia-Romagna and Veneto. The Covid-19 CI has remained substantially lower in the central and southern regions, as exemplified here by the curves for Tuscany, Campania and Sicily. The figure shows the modelled curves superimposed to the observed data, thus indicating an overall good fit.

Based on this model, we calculated the evolution of the Covid-19 CI growth rate for the same six regions (Fig. 1b). We found that a peak was firstly reached in Lombardy the 22nd of March, followed by Emilia-Romagna and Veneto the 24th of March, Sicily the 25th of March, Tuscany the 26th and Campania the 27th of March. These results indicate that the time lag between the introduction of the government measures, the 9th and 12th of March, and the peaking of the Covid-19 CI growth rate was between 13 and 15 days. The analyses of the curves of these six regions also indicate that at the time the government measures were introduced, the epidemic was at very different stages across these regions. The evolution of the epidemic curves suggests that the government measures achieved a reduction and a eventually a peaking of the Covid-19 CI growth rate both in the Northern Italian regions, where the epidemic was already rampant around the 9–11 March, as well as in central and southern Italy where the epidemic curve was still low at that time. The results also suggest that the government measures may have prevented the Covid-19 epidemic in central and southern regions to rise to the high levels that were already occurring in the North.

**Fig. 2:**
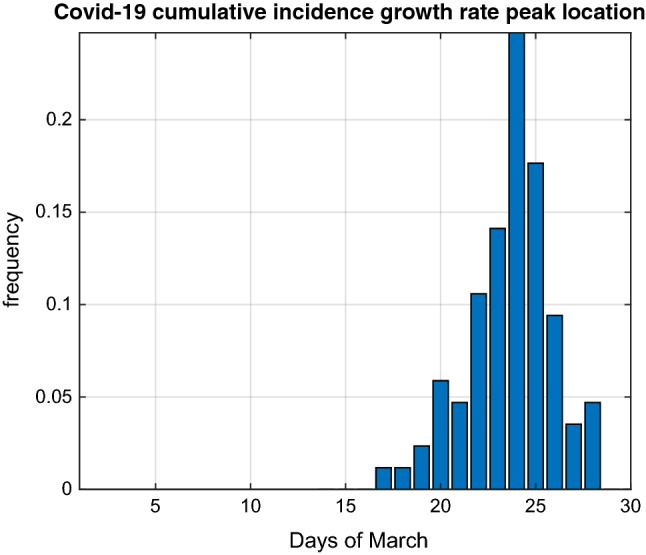
Observed distribution of the day of March 2020 when 87 Italian provinces reached a peak of the Covid-19 cumulative incidence growth rate between the 1st and the 31st of March. The 21 out of a total of 107 provinces that had not reached a peak by the 31st of March, are not represented in the histogram

In Fig. 2 we report the distribution of the day when each province reached a peak of the Covid-19 CI growth rate. At the time of conducting our analyses, the 31st of March 2020, 87 provinces out of 107 had reached a peak and their daily distribution indicates that the mode was reached on the 24th March with 21 provinces reaching a peak that day. The mean value of time for reaching the peak was 23.6 days starting from the 1st of March and 12.6 days since the extension to the whole country of the restriction  measures.

**Fig. 3:**
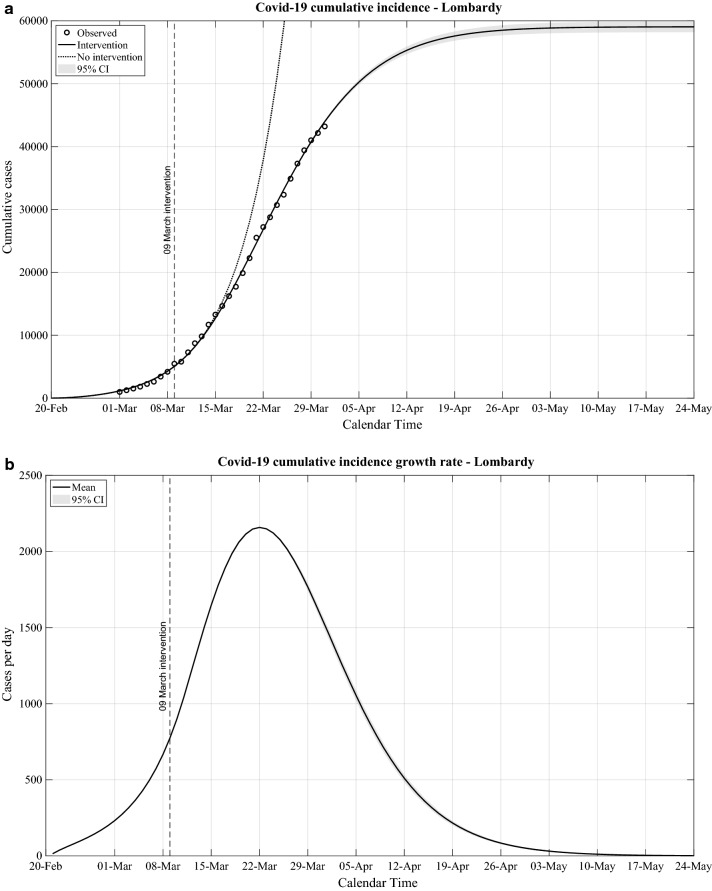
Covid-19 epidemic evolution in Lombardy. **a:** Observed cumulative number of cases from the 1st to the 31st March 2020 and estimated evolution of the cumulative incidence by the proposed Bayesian approach; **b:** estimated cumulative incidence growth rate

We further analysed the evolution of the Covid-19 CI and estimated the projected evolution using a model that takes into account the governmental measures. In addition, we estimated the Covid-19 CI curve that would have been observed assuming no government interventions. In Fig. 3a we report the results for Lombardy, as the region where the epidemic reached by far the highest Covid-19 CI. The figure shows that the model follows closely the observed Covid-19 CI data. Furthermore, our results indicate that the Covid-19 CI progressively diverged toward values lower than the ones expected by the projected evolution of the epidemic in the absence of government measures. The two curves started to separate approximately 7–10 days after the implementation in Lombardy of the government measures the 9th of March 2020. This estimated time interval is in line with the one obtained as the sum of the 5 days median interval between infection and symptoms [5] and the 4 days median interval between onset of symptoms and diagnoses as reported in Italy [6].

Based on the projected evolution of the Covid-19 CI to the 24th of May 2020, we calculated the projection of its growth rate (Fig. 3b). We estimated that the epidemic in Lombardy should substantially slow down towards mid-May 2020 and it should reduce to the level of sporadic cases by end of May 2020. The projected evolution is based on a number of assumptions, namely that the current measures would remain effectively implemented during the entire period covered by our analyses and that there will be no influx of Covid-19 cases from external areas. Using the same model, we analysed the data of other Italian regions, and observed similar patterns in Emilia-Romagna, Veneto, Tuscany, Campania and Sicily with a substantial reduction of this epidemic wave within May 2020 (data available on request).

## Discussion

The results we present here provide evidence that the strict measures implemented in Lombardy and surrounding areas and shortly thereafter extended to the whole of Italy have made a measurable impact in reducing the progression of the Covid-19 epidemic. We estimated that the time lag between the start of the implementation of the restriction measures and the measurable reduction of the Covid-19 CI growth rate was approximately 7–10 days.

We also found that, by the 31st of March 2020, the epidemic growth rate reached a peak in 87 out of 107 Italian provinces, confirming that the epidemic is now in decline. Our projections of the evolution of the epidemic curve suggest that Lombardy and most Italian regions should reach the final stage of this epidemic wave within May 2020, with only sporadic cases expected to occur thereafter. These projections assume that the government measures will remain in place during this entire period.

The comparison of the curves of the epidemic in different Italian regions in relation to the time when the government measures were introduced (9th to 12th of March 2020), suggests that the earlier the measures were taken in relation to the phase of the epidemic in that particular region, the lower the cumulative incidence achieved during this epidemic wave. These observations indicate that the government measures were effective to both slow down the epidemic that was rampant in the North of Italy and prevent the epidemic in the centre and south of Italy the rise to the detrimental levels that were already present in the North of Italy in mid-March 2020.

At the time when several governments indicate that their aim  is to “push down” the epidemic curve, the results of the Italian regions support the WHO recommendation that strict containment measures should be introduced as early as possible in the epidemic curve.
